# Systemic immune-inflammation index predicting chemoradiation resistance and poor outcome in patients with stage III non-small cell lung cancer

**DOI:** 10.1186/s12967-017-1326-1

**Published:** 2017-10-31

**Authors:** Yu-Suo Tong, Juan Tan, Xi-Lei Zhou, Ya-Qi Song, Ying-Jian Song

**Affiliations:** 1 Department of Radiation Oncology, Huai’an First People’s Hospital, Nanjing Medical University, Huai’an, Jiangsu China; 2 Department of Gerontology, Huai’an First People’s Hospital, Nanjing Medical University, Huai’an, Jiangsu China; 3 Department of Respiratory Medicine, Huai’an First People’s Hospital, Nanjing Medical University, Huai’an, Jiangsu China

**Keywords:** Non-small cell lung cancer, prognostic factor, Systemic immune-inflammation index, Chemoradiation

## Abstract

**Background:**

There is increasing evidence that the existence of systemic inflammation response is correlated with poor prognosis in several solid tumors. The aim of this retrospective study was to investigate the association between systemic immune-inflammation index (SII) and therapy response and overall survival in patients with stage III non-small cell lung cancer (NSCLC). The prognostic values of neutrophil to lymphocyte ratio (NLR), platelet to lymphocyte ratio (PLR), and prognostic nutritional index (PNI) were also evaluated.

**Methods:**

In total, 332 patients with new diagnosis of stage III NSCLC were included in this retrospective analysis. SII was defined as platelet counts × neutrophil counts/lymphocyte counts. Receiver operating characteristic (ROC) curve was used to evaluate the optimal cut-off value for SII, NLR, PLR and PNI. Univariate and multivariate survival analysis were performed to identify the factors correlated with overall survival.

**Results:**

Applying cut-offs of ≥ 660 (SII), ≥ 3.57 (NLR), ≥ 147 (PLR), ≤ 52.95 (PNI), SII ≥ 660 was significantly correlated with worse ECOG PS (< 0.001), higher T stage (< 0.001), advanced clinical stage (*p* = 0.019), and lower response rate (*p* = 0.018). In univariate analysis, SII ≥ 660, NLR ≥ 3.57, PLR ≥ 147, and PNI ≤ 52.95 were significantly associated with worse overall survival (*p*_all_ < 0.001). Patients with SII ≥ 660 had a median overall survival of 10 months, and patients with SII < 660 showed a median overall survival of 30 months. In multivariate analysis only ECOG PS (HR, 1.744; 95% CI 1.158–2.626; *p* = 0.008), T stage (HR, 1.332; 95% CI 1.032–1.718; *p* = 0.028), N stage (HR, 1.848; 95% CI 1.113–3.068; *p* = 0.018), SII (HR, 2.105; 95% CI 1.481–2.741; *p* < 0.001) and NLR ≥ 3.57 (HR, 1.934; 95% CI 1.448–2.585; *p* < 0.001) were independently correlated with overall survival.

**Conclusions:**

This study demonstrates that the SII is an independent prognostic indicator of poor outcomes for patients with stage III NSCLC and is superior to other inflammation-based factors in terms of prognostic ability.

## Background

Lung cancer remains the leading cause of cancer-related mortality in the world, accounting for 1.3 million deaths each year [1]. Non-small cell lung cancer (NSCLC) compromises more than 85% of all lung cancers cases [2]. Approximately 20–25% patients with NSCLC are diagnosed with locally advanced disease (stage III) and have poor survival [3]. For these patients, two standard treatment options are offered: the concurrent chemoradiotherapy (CRT) or induction chemotherapy followed by surgery [4, 5]. However, even after complete resection and postoperative consolidation chemotherapy, 20–40% patients still have a risk of local recurrence [6]. Indeed, NSCLC is poorly chemosensitive to most of the available agents, the reported treatment response rates is only 10–25% [7]. Chemotherapy resistance and development of local recurrence or distant metastases are the main obstacles in the treatment of locally advanced NSCLC. Therefore, identification of prognostic factors that can be used to predict treatment response or long-term survival is required.

It is widely recognized that systemic inflammation plays an important role in the development and progression of many solid tumors [8]. The existence of systemic inflammation, as measured by parameters such as neutrophil to lymphocyte ratio (NLR), platelet to lymphocyte ratio (PLR), and prognostic nutritional index (PNI) was reported to be correlated with poor prognosis across multiple malignancies, including NSCLC [9–13]. Recently, the systemic immune-inflammation index (platelet counts × neutrophil counts/lymphocyte counts, SII) has been shown to have independent prognostic value in patients with hepatocellular carcinoma treated with surgery [14]. However, the clinical implication of SII in chemoradiotherapy resistance and survival in locally advanced NSCLC remains largely unknown.

The present study had three aims: first, to evaluate the prognostic significance of SII in patients with locally advanced NSCLC treated with primary chemoradiotherapy, second, to investigate whether SII was able to predict treatment response to chemoradiotherapy. Finally, to compare the prognostic values of inflammation-based prognostic factors (NLR, PLR, and PNI) with SII.

## Methods

### Patient section

The study was approved by the medical ethics committee of our institute, and informed consent was exempted due to the retrospective nature of the study.

Consecutive patients who were newly diagnosed with stage III NSCLC between January 2006 and May 2012 in our hospital (Affiliated Huai’an First Hospital, Nanjing Medical University, Jiangsu, China) were collected in the present study. All medical records were reviewed retrospectively. Patients who met the following inclusion criteria were selected: (a) biopsy proven NSCLC; (b) stage III A or stage III B disease according to the 6th edition of tumor-node-metastasis (TNM) classification; (c) Eastern Cooperative Oncology Group performance status (ECOG PS) (0–2); (d) ≤ 70 years of age; (e) treatment with concurrent CRT or surgery followed by chemoradiotherapy. Patients with hematologic malignancies, chronic inflammatory disease, or clinical evidence of acute infection were excluded. Patients who received neoadjuvant chemotherapy were also excluded.

### Data collection and definition

Patient characteristics including age, sex, history of tobacco exposure, pathologic type, TNM stage, full blood count, ECOG PS and the details of treatment were collected by electronic medical reports. Full blood counts were obtained before the initiation of any treatment (surgery, radiation, or chemotherapy). The SII, NLR, PLR and PNI were calculated as follows: SII = platelet counts × neutrophil counts/lymphocyte counts, NLR = neutrophil count/lymphocyte count, PLR = platelet count/lymphocyte count, PNI = albumin (g/L) + 5 × total lymphocyte count (10^9^/L).

### Treatment details

#### Surgery-chemoradiotherapy group

All patients underwent tumor resection and systemic lymph node dissection. 4–6 weeks after surgery, cisplatin-based adjuvant chemotherapy was performed every 3 weeks. 2–4 cycles of chemotherapy were administered according the decisions of the physicians. If there was incomplete resection (RI or R2) after surgery, patients received postoperative radiotherapy (1.8–2.0 Gy/day, 5 days/week, 50.4–66 Gy).

#### CRT group

Concurrent radiotherapy using 6 or 15 MV X-rays was delivered at a dose of 1.8–2.0 Gy/day, 5 days/week, with a total radiation dose of 40–66 Gy. Chemotherapy started on day 1, concurrent with the beginning of radiation. Most patients received four to six cycles of cisplatin-based concurrent chemotherapy.

### Response assessment and follow-up

One month after complete of treatment, tumor response was evaluated by CT scan according to the Response Evaluate Criteria for Solids Tumors (RECIST). Complete response (CR) was defined as total regression of all assessable lesions; partial response (PR) was defined as the disappearance of at least of 30% in the sum of the longest diameters of the target lesions; progressive disease (PD) was defined as more than a 20% increase in primary tumor volume or appearance of new lesions; the remaining patients which did not meet the criteria of PD or PR were categorized as stable disease (SD) [15]. The objective response rates were calculated by the percentage of CR and PR among all treated patients.

Patients were followed every 3 months for the first year, then every 6 months for 2 years, and then every year or until death. Follow-up data were obtained from patient medical records and telephone interview.

### Statistical analysis

All statistical analyses were performed using SPSS software (version 20.0). Receiver operating characteristic (ROC) curves were used to calculate the optimal cut-off value for SII, NLR, PLR and PNI, and the end-point was based on overall survival (OS) in the study. Categorical variables were reported as frequencies and percentages, and were analyzed using Fisher’s exact tests or Chi square tests. OS was defined as the time from diagnosis to date of death due to any cause. Data from patients who were alive by the time of analysis were censored. Survival analyses were performed using Kaplan–Meier method. The differences between the survival curves were compared by using Log rank test. The multivariate Cox hazard regression analysis was performed on the factors that were shown to be significant on univariate analysis. All tests were two-sided and *p* values less than 0.05 were considered significant.

## Results

### Patient characteristics

Between January 2006 and May 2012, a total of 545 patients with stage III NSCLC were initially identified, of whom 332 patients were eligible for analysis. Of the included patients, 115 (35%) patients underwent surgical resection followed by chemotherapy or chemoradiotherapy, the remaining patients (n = 217, 65%) received concurrent CRT. Baseline patient characteristics are summarized in Table 1. The median age was 61 (range 34–70) years, 206 (62%) patients were male and 126 (38%) were female. Among these patients, 197 (59.3%) had stage IIIA UICC-6 disease, and 135 (40.7%) had stage IIIB UICC-6 disease. Approximate half of the patients (n = 154, 46.4%) were determined to have adenocarcinoma, 161 (48.5%) had squamous cell carcinoma, 17 (5.1%) had adenosquamous carcinoma, or other histology.

**Table 1:** Baseline patient characteristics

Characteristics	Number (%)
Age	
Median	61
Range	34–70
Gender	
Male	206 (62%)
Female	126 (38%)
Smoking status	
Never smoker	141 (42.5%)
Current or ex-smoker	191 (57.5%)
ECOG PS	
0–1	304 (91.6%)
2	28 (8.4%)
Histological subtype	
Adenocarcinoma	154 (46.4%)
Squamous	161 (48.5%)
Other histology	17 (5.1%)
T stage	
T1	59 (17.8%)
T2	122 (36.7%)
T3	74 (22.3%)
T4	77 (23.2%)
N stage	
N0	24 (7.2%)
N1	29 (8.7%)
N2	221 (66.6%)
N3	58 (17.5%)
Clinical stage	
III A	197 (59.3%)
III B	135 (40.7%)
Treatment modality	
Surgery + chemoradiation	115 (34.6%)
Concurrent chemoradiation	217 (65.4%)
Chemotherapy cycles	
Median	4
Range	2–8
Chemotherapy regimen utilized	
Cisplatin + etoposide	36 (10.8%)
Cisplatin + docetaxel	150 (45.2%)
Cisplatin + paclitaxel	61 (18.4%)
Cisplatin + vinorebine	47 (14.2%)
Cisplatin + others	38 (11.4%)

In the surgery-chemoradiotherapy group, all patients underwent surgical resection followed by cisplatin-based doublet chemotherapy. The median number of chemotherapy cycle was four (range 2–4 cycles). For patients with R1 or R2 resection, postoperative radiation therapy was administered in 47 patients. In the concurrent CRT group, all patients received cisplatin-based concurrent chemotherapy, including cisplatin in combination with etoposide (n = 23), cisplatin plus docetaxel (n = 96), cisplatin plus paclitaxel (n = 54) and cisplatin plus vinorebine (n = 44). After concurrent CRT, 171 patients (78.8%) revived four courses of consolidation chemotherapy. The most commonly used chemotherapy regimen for consolidation was docetaxel and cisplatin.

At baseline, the median values of SII, NLR, PLR and PNI for all study population were 634.14 (range 159.80–4299.90), 3.05 (range 0.59–19.28), 141.29 (range 34.63–571.79) and 50.31 (range 15.59–67.75), respectively.

### Tumor response to CRT and patient outcomes

Of the 217 patients who received concurrent CRT, CR, PR, SD and PD were observed in 12 (5.5%), 137 (63.2%), 64 (29.5%) and 4 (1.8%) cases, respectively. The objective response rates were 68.7% (149/217).

With a median follow-up time of 22 months (range 2–72 months), 281 (84.6%) patients had died and 51 (15.4%) patients were living at the end of the follow-up period. The 1-, 3- and 5-year OS of all study population were 65.4, 28.6, 15.4%, respectively.

### Selection of optimal cut-off values for SII, NLR, PLR, and PNI

**Fig. 1:**
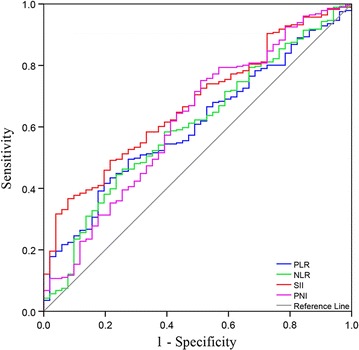
Receiver operating characteristic curve analysis for the optimal cut-off value of SII, NLR, PLR and PNI. The areas under the curve for overall survival were 0.673, 0.603, 0.604 and 0.621 for SII, NLR, PLR and PNI, respectively

Different studies have suggested different cut-off values when analyzing SII, NLR, PLR and PNI in prognostic setting. We therefore attempted to establish the optimal thresholds for these biomarkers on our study population through ROC curve analysis. As shown in Fig. 1, the area under the curves (AUC) for OS were 0.673 (*p* < 0.001), 0.604 (*p* = 0.019), 0.603 (*p* = 0.018) and 0.621 (*p* = 0.006) for SII, NLR, PLR and PNI, respectively. The optimal cut-off values for the prediction of OS by ROC analysis was 660 for SII, 3.57 for NLR, 147 for PLR and 52.95 for PNI. Consequently, patients were separately divided into two groups with high or low levels according to the optimal cut-off values. One hundred and forty-nine patients (44.9%) had SII ≥ 660, 137 patients (41.3%) had NLR ≥ 3.57, 153 patients (46.1%) had PLR ≥ 147 and 94 (28.3%) patients had PNI ≥ 52.95.

### Association of inflammation-based factors with clinicopathological characteristics

The relationship between SII, NLR, PLR and PNI and patient characteristics is shown in Table 2. Using the criteria described earlier, patients with SII ≥ 660 were more likely to have worse ECOG PS (*p* < 0.001), higher tumor (T) stage (*p* < 0.001) and advanced clinical stage (*p* = 0.019) than those with SII < 660. In addition, patients with high NLR and PLR had statistically significantly advanced clinical stage compared those with low NLR and PLR (*p* = 0.035 and < 0.001, respectively). Patients with tobacco use also had higher NLR (*p* = 0.006). By contrast, patients with high PNI were significantly younger (*p* = 0.037) and have better ECOG PS (*p* = 0.031) than those with low PNI. Moreover, PNI was significantly higher in patients diagnosed with stage III A cancer compared with those with stage III B disease (*p* = 0.022).

**Table 2:** Clinicopathological characteristic according SII, NLR, PLR and PNI

Characteristics	SII ≥ 660 n = 149	SII < 660 n = 183	*p*	NLR ≥ 3.57 n = 137	NLR < 3.57 n = 195	*p*	PLR ≥ 147 n = 153	PLR < 147 n = 179	*p*	PNI ≥ 52.95 n = 94	PNI < 52.95 n = 238	*p*
Age			0.145			0.413			0.147			0.037
< 60	67 (45%)	97 (53%)		64 (47%)	100 (51%)		69 (45%)	95 (53%)		55 (59%)	109 (46%)	
≥ 60	82 (55%)	86 (47%)		73 (53%)	95 (49%)		84 (55%)	84 (47%)		39 (41%)	129 (54%)	
Gender			0.725			0.647			0.372			0.181
Male	94 (63%)	112 (61%)		87 (64%)	119 (61%)		91 (59%)	115 (64%)		53 (56%)	153 (64%)	
Female	55 (37%)	71 (39%)		50 (36%)	76 (39%)		62 (41%)	64 (36%)		41 (44%)	85 (36%)	
Smoking status			0.239			0.006			0.996			0.448
No	58 (39%)	83 (45%)		46 (34%)	95 (49%)		65 (42%)	76 (42%)		43 (46%)	98 (41%)	
Yes	91 (61%)	100 (55%)		91 (66%)	100 (51%)		88 (58%)	103 (58%)		51 (54%)	140 (59%)	
ECOG PS			< 0.001			0.167			0.105			0.031
0–1	127 (85%)	177 (97%)		122 (89%)	182 (93%)		136 (89%)	168 (94%)		91 (97%)	213 (89%)	
2	22 (15%)	6 (3%)		15 (11%)	13 (7%)		17 (11%)	11 (6%)		3 (3%)	25 (11%)	
Histology			0.508			0.863			0.778			0.384
Adenocarcinoma	74 (50%)	80 (44%)		64 (47%)	90 (46%)		74 (48%)	80 (45%)		38 (40%)	116 (49%)	
Squamous	67 (45%)	94 (51%)		65 (47%)	96 (49%)		71 (46%)	90 (50%)		51 (54%)	110 (46%)	
Other histology	8 (5%)	9 (5%)		8 (6%)	9 (5%)		8 (6%)	9 (5%)		5 (6%)	12 (5%)	
T stage			< 0.001			< 0.001			0.063			0.245
T1–T2	58 (39%)	123 (67%)		52 (38%)	129 (66%)		75 (49%)	106 (59%)		56 (60%)	125 (53%)	
T3–T4	91 (61%)	60 (33%)		85 (62%)	66 (34%)		78 (51%)	73 (41%)		38 (40%)	113 (47%)	
N stage			0.108			0.211			0.084			0.014
Negative	7 (5%)	17 (9%)		7 (5%)	17 (9%)		7 (5%)	17 (9%)		12 (13%)	12 (5%)	
Positive	142 (95%)	166 (91%)		130 (95%)	178 91(%)		146 (95%)	162 (91%)		82 (87%)	226 (95%)	
Clinical stage			0.019			0.035			< 0.001			0.022
III A	78 (52%)	119 (65%)		72 (53%)	125 (64%)		74 (48%)	123 (69%)		65 (69%)	132 (55%)	
III B	71 (48%)	64 (35%)		65 (47%)	70 (36%)		79 (52%)	56 (31%)		29 (31%)	106 (45%)	
Response			0.018			0.877			0.151			0.715
CR + PR	73 (62%)	76 (77%)		74 (69%)	75 (68%)		72 (64%)	77 (73%)		36 (67%)	113 (69%)	
SD + PD	45 (38%)	23 (23%)		33 (31%)	35 (32%)		40 (36%)	28 (27%)		18 (33%)	50 (31%)	

*CR* complete response, *PR* partial response, *PD* progressive disease, *SD* stable disease, *SII* systemic immune-inflammation index, *NLR* neutrophil/lymphocyte ratio, *PLR* platelet/lymphocyte ratio, *PNI* prognostic nutritional index

### Baseline SII and response to treatment

A total of 217 patients underwent concurrent CRT. In patients who were SII ≥ 660 (n = 118, 54.4%), CR, PR, SD and PD were observed in 3 (2.6%), 70 (59.3%), 43 (36.4%) and 2 (1.7%) cases, respectively. However, in patients with SII < 660 (n = 99, 45.6%), CR, PR, SD and PD were achieved in 9 (9.1%), 67 (67.7%), 21 (21.2%) and 2 (2%) patients, respectively. Thereafter, patients with SII < 660 had significantly higher response rate to treatment than those with SII ≥ 660 (76.8% vs 61.9%, *p* = 0.018). However, NLR, PLR, and PNI did not show any significant correlation with treatment response (*p*_all_ > 0.05).

### Prognostic value of SII, NLR, PLR and PNI and other clinicopathological factors

**Fig. 2:**
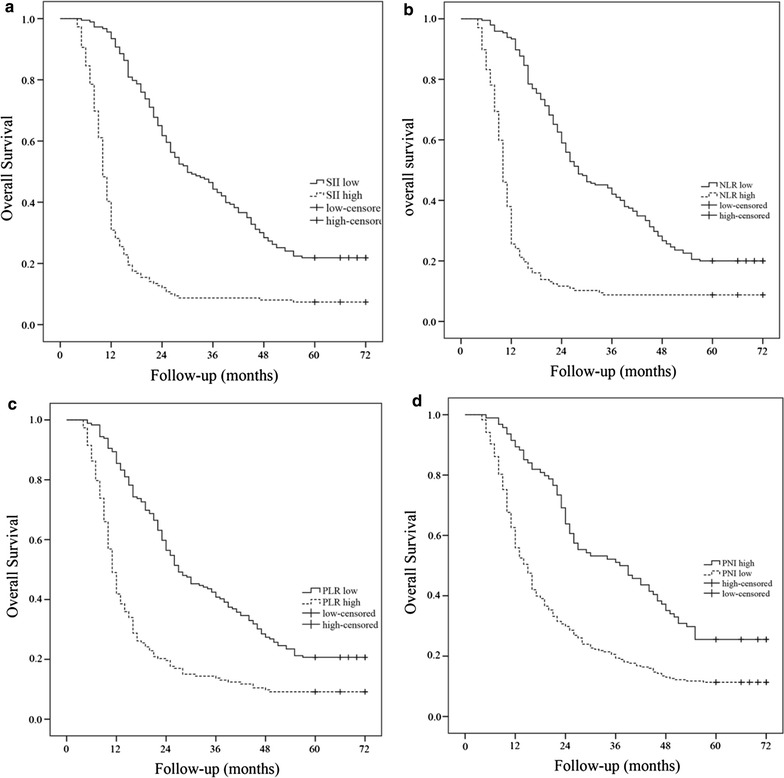
Kaplan–Meier curves of overall survival according to SII (**a**), NLR (**b**), PLR (**c**) and PNI (**d**)

The correlation between inflammation-based factors and OS is shown in Fig. 2. Patients with SII ≥ 660, NLR ≥ 3.57, PLR ≥ 147 and PNI < 52.95 had significantly worse OS (*p*_all_ < 0.05). Patients with SII ≥ 660 had a median OS of 10 months whereas patients with SII < 660 showed a median OS of 30 months. In addition, patients with NLR ≥ 3.57 had a median OS of 10 months, compared with 28 months for patients with NLR < 3.57. The median OS was 11 months for patients with PLR ≥ 147 and 27 months for patients with PLR < 147. And the median OS was 15 months for patients with PNI < 52.95 and 27 months for patients with PNI ≥ 52.95. SII provided the greatest survival difference with a 5-year OS rate of 21.9% in SII < 660 vs 7.4% in SII ≥ 660, followed by PNI (25.5% PNI ≥ 52.95 vs 11.3% PNI < 52.95), and then PLR (20.7% PLR < 147 vs 9.2% PLR ≥ 147), and NLR (20% NLR < 3.57 vs 8.8% NLR ≥ 3.57).

**Fig. 3:**
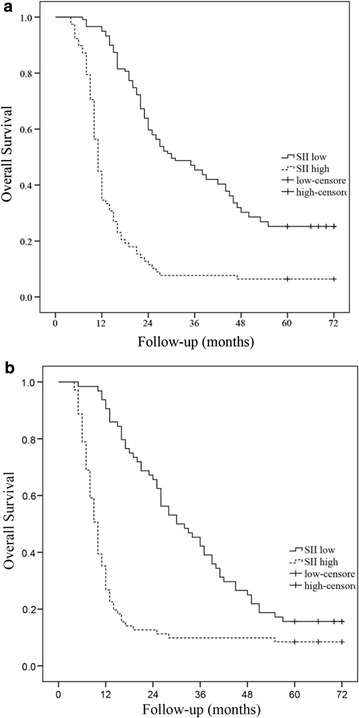
Kaplan–Meier survival analysis in stage III A (**a**) and stage III B (**b**) NSCLC subgroup

However, in this analysis, SII ≥ 660 group contained many patients (47.7%) who underwent concurrent CRT for stage III B disease compared with patients in SII < 660 group (30.3%), which may influence the results. The prognostic value of SII was next investigated in stage III A and stage III B subgroup, separately. As shown in Fig. 3, patients with SII ≥ 660 still have worse OS in both stage III A and stage III B disease. Separate analyses the prognostic value of SII, NLR, PLR and PNI in patients with adenocarcinoma and squamous cell carcinoma also showed significant effects in both groups (*p*_all_ < 0.05, data not shown).

On univariate cox regression analyses, ECOG PS (*p* < 0.001), T stage (*p* = 0.002), node (N) stage (*p* = 0.028), clinical stage (*p* = 0.034), SII (*p* < 0.001), NLR (*p* < 0.001), PLR (*p* < 0.001) and PNI (*p* < 0.001) were significantly correlated with OS (Table 3). All 8 clinicopathological characteristics were further investigated in multivariate analysis. As shown in Table 4, SII (HR, 2.105; 95% CI 1.481–2.741; *p* < 0.001) was the most significantly independent predictor of worse OS, followed by NLR (HR, 1.934; 95% CI 1.448–2.585; *p* < 0.001). Meanwhile, ECOG PS (HR, 1.744; 95% CI 1.158–2.626; *p* = 0.008), T stage (HR, 1.332; 95% CI 1.032–1.718; *p* = 0.028) and N stage (HR, 1.848; 95% CI 1.113–3.068; *p* = 0.018) were also independent risk factors for OS.

**Table 3:** Univariate analysis of potential factors associated with overall survival in patients with locally advanced NSCLC

Variables	Case	Overall survival	p*
		Median (months)	
Age			0.691
< 60	164	21	
≥ 60	168	17	
Gender			0.492
Male	206	18	
Female	126	19	
Smoking status			0.154
Never smoker	141	21	
Current or ex-smoker	191	16	
ECOG PS			< 0.001
0–1	304	21	
2	28	12	
Histological subtype			0.654
Adenocarcinoma	154	18	
Squamous	161	21	
Other histology	17	14	
T stage			0.002
T1–T2	181	22	
T3–T4	151	14	
N stage			0.028
Negative	24	42	
Positive	308	17	
Clinical stage			0.034
III A	197	21	
III B	135	15	
Chemotherapy cycles			0.741
< 4	72	16	
≥ 4	260	19	
SII			< 0.001
< 660	183	30	
≥ 660	149	10	
NLR			< 0.001
< 3.57	195	28	
≥ 3.57	137	10	
PLR			< 0.001
< 147	179	27	
≥ 147	153	11	
PNI			< 0.001
≥ 52.95	94	36	
< 52.95	238	15	

*CR* complete response, *PR* partial response, *PD* progressive disease, *SD* stable disease, *SII* systemic immune-inflammation index, *NLR* neutrophil/lymphocyte ratio, *PLR* platelet/lymphocyte ratio, *PNI* prognostic nutritional index

* *p* log-rank test

**Table 4:** Multivariate analysis of potential factors associated with overall survival in patients with locally advanced NSCLC

Variables	HR	95% CI	*p*
ECOG PS			0.008
0–1	1.00		
2	1.744	1.158–2.626	
T stage			0.028
T1–T2	1.00		
T3–T4	1.332	1.032–1.718	
N stage			0.018
Negative	1.00		
Positive	1.848	1.113–3.068	
Clinical stage			0.653
III A	1.00		
III B	1.058	0.828–1.350	
SII			< 0.001
< 660	1.00		
≥ 660	2.105	1.481–2.741	
NLR			< 0.001
< 3.57	1.00		
≥ 3.57	1.934	1.448–2.585	
PLR			0.083
< 147	1.00		
≥ 147	1.299	0.966–1.748	
PNI			0.131
≥ 52.95	1.00		
< 52.95	1.263	0.933–1.709	

*HR* hazard ratio, *95 CI* 95% confidence interval, *SII* systemic immune-inflammation index, *NLR* neutrophil/lymphocyte ratio, *PLR* platelet/lymphocyte ratio, *PNI* prognostic nutritional index

## Discussion

In the present study, we evaluated prognostic value of inflammation-based factors (SII, NLR, PLR and PNI) in patients with stage III NSCLC treated with primary chemoradiotherapy to identify patients who could benefit from current treatment. We found that patients with SII ≥ 660 were more likely to have higher T stage, worse ECOG PS, advanced clinical stage and lower response rate than patients with SII < 660. Furthermore, the pre-treatment SII was also found to be an independent prognostic biomarker for OS and was superior to NLR, PLR and PNI in terms of prognostic ability.

Recently, several studies have revealed that inflammation-based factors are correlated with aggressive tumor characteristics in various tumors. In the study by Deng et al., NLR and PLR were significantly associated with tumor stage, deep of invasion, and lymph node metastasis in patients with gastric cancer [16]. In another study of 112 patients with hepatocellular carcinoma, patients with PNI < 45 were more likely to have portal vein thrombosis and worse Child-Turcotte-Pugh class [17]. In consistent with these earlier results, SII ≥ 660 were 60% in T3/4 cases compared with 32% in T1/2 cases in our results.

Although cisplatin-based concurrent CRT has been the standard treatment option for locally advanced NSCLC, chemotherapy resistance remains the main obstacle in cancer treatment [18, 19]. Chronic inflammation plays an important role in induction of chemoradiation resistance. In this study, we observed that high SII was associated with chemoradiation resistance in patients with locally advanced NSCLC. Several inflammation-based biomarkers are known to be correlated with treatment response. A recent retrospective study found that high NLR was significantly correlated with chemotherapy resistance in patient with advanced NSCLC treated with first line platinum-based chemotherapy [20]. Similar findings have also been observed by Cho and Mabuchi that tumor-related leukocytosis (TRL) was significantly correlated with poor radiation response in patient with uterine cervical carcinoma [21, 22]. In the retrospective analysis by Mabuchi, TRL (+) patients had upregulated tumor granulocyte colony-stimulating factor (G-CSF) and increased myeloid-derived suppressor cells (MDSCs) in blood. MDSCs are heterogenic and immunosuppressive subpopulation of cells that enhance tumor progression through stimulating vasculogenesis [23]. These results suggested that G-CSF-induced MDSCs stimulated angiogenesis may partly contribute to tumor resistance to radiation.

The prognostic significances of inflammation-based biomarkers have been shown in many solid tumors, most notably in prostate cancer, colorectal cancer, esophageal squamous cell carcinoma, melanoma and NSCLC [24–28]. Like others, we have demonstrated that baseline SII ≥ 660, NLR ≥ 3.57, PLR ≥ 147 and PNI < 52.95 could predict poor clinical outcomes for the whole cohort of patient with locally advanced NSCLC. SII and NLR remained significantly prognostic even after adjusted for other parameters, such as T stage, N stage, ECOG performance status and clinical stage. Moreover, our multivariate analysis showed that SII was superior to NLR in terms of prognostic ability. Our study is not the first to assess SII and cancer patient prognosis. Hu et al. have previously demonstrated the prognostic significance of SII in patients with hepatocellular carcinoma receiving curative resection [14]. The mechanism by which high SII contributes to a poor prognosis in patients with locally advanced NSCLC is unclear. Theories at present focus on the relative neutrophilia, thrombocytosis and lymphopenia that occur as part of systemic inflammation response triggered by cancer. In the present study, SII was calculated as platelet counts × neutrophil counts/lymphocyte counts. Patients with elevated SII often have thrombocytosis, neutrophilia, and/or lymphopenia. Thrombocytosis, a paraneoplastic syndrome, has been reported in as many as 10–57% of cancer patients [29]. Recent studies have found a significant role of platelets during tumor development and progression. Indeed, there is increasing evidence that a high level of platelet count is associated with worse survival in patients with cancer [30]. An elevated platelet counts could stimulate tumor angiogenesis and protect tumor cells from cytolysis, thereby contributing to tumor metastasis [31]. Furthermore, neutrophils have also been shown to have tumor-promoting abilities. Relative neutrophilia increases the number of inflammatory factors such as pro-angiogenic factor (VEGF), growth factor (CXCL8), and anti-apoptotic factor (NF-κB) which may establish a tumor microenvironment and promote tumor growth and progression [32, 33]. In contrast to neutrophils, lymphocytes have an important role in tumor defence by inducing cytotoxic cell death and inhibiting tumor cell proliferation and migration [34, 35]. A relative lymphopenia may reflect a lower number of CD4^+^ T helper lymphocytes, resulting in a poorer lymphocyte-mediated immune response to malignancies [36]. All of these may promote tumor cells growth, progression and metastasis.

The present study has several limitations despite the demonstration of the prognostic value of SII in patients with stage III NSCLC. First, this is a retrospective analysis; hence there are several potential factors that might have influenced the studied results. Second, data on all patients were collected from a single institute and number of patients is relatively small. Also, the section of treatment modalities and chemotherapy regimens were heterogeneous throughout the period. Therefore, a multi-institutional investigation, especially a prospective validation study is needed to confirm the results.

## Conclusions

In conclusion, our study has demonstrated that the SII, an inflammation-based prognostic biomarker, is an independent prognostic marker for poor survival in patients with stage III NSCLC. In particular, SII is superior to NLR, PLR and PNI in terms of prognostic ability.

## Abbreviations

NSCLC: non-small cell lung cancer
CRT: chemoradiotherapy
NLR: neutrophil to lymphocyte ratio
PLR: platelet to lymphocyte ratio
PNI: prognostic nutritional index
SII: systemic immune-inflammation index
TNM: tumor-node-metastasis
ECOG PS: Eastern Cooperative Oncology Group performance status
RECIST: Response Evaluation Criteria in Solid Tumor
CR: complete response
PR: partial response
PD: progressive disease
SD: stable disease
ROC: receiver operating characteristic
OS: overall survival
TRL: tumor-related leukocytosis
MDSCs: myeloid-derived suppressor cells
HR: hazard ratio
CI: confidence interval
